# A Test for Glycerin

**Published:** 1869-09

**Authors:** 


A Test for Glycerin.—The increased use for glycerin in the arts of late has, of course, brought into the market an adulterated article. When sugar and dextrine were mixed in small proportions with glycerin it has hitherto been difficult to detect the adulteration, but is now easily done by the following method: To five drops of the glycerin to be tested add 100 to 120 drops of water, one drop of pure nitric acid, and three to four centigrammes of ammonium molybdate, and boil the mixture, and in less than two minutes it will assume a deep blue color if any sugar or dextrine is present.

